# Identification of let-7f and miR-338 as plasma-based biomarkers for sporadic amyotrophic lateral sclerosis using meta-analysis and empirical validation

**DOI:** 10.1038/s41598-022-05067-4

**Published:** 2022-01-26

**Authors:** Narges Daneshafrooz, Mohammad Taghi Joghataei, Mehdi Mehdizadeh, Afagh Alavi, Mahmood Barati, Bahman Panahi, Shahram Teimourian, Babak Zamani

**Affiliations:** 1 Cellular and Molecular Research Center, Department of Neuroscience, Faculty of Advanced Technologies in Medicine, Iran University of Medical Science, Tehran, Iran; 2 Reproductive Sciences and Technology Research Center, Department of Anatomy, School of Medicine, Iran University of Medical Science, Tehran, Iran; 3 Genetics Research Center, University of Social Welfare and Rehabilitation Sciences, Tehran, Iran; 4 Department of Medical Biotechnology, Faculty of Allied Medicine, Iran University of Medical Science, Tehran, Iran; 5 Department of Genomics, Branch for Northwest & West Region, Agricultural Biotechnology Research Institute of Iran (ABRII), Agricultural Research, Education and Extension Organization (AREEO), Tabriz, Iran; 6 Department of Medical Genetics, School of Medicine, Iran University of Medical Science, Tehran, Iran; 7 Department of Neurology, Firoozgar Hospital, School of Medicine, Iran University of Medical Science, Tehran, Iran

**Keywords:** Molecular biology, Neuroscience, Biomarkers, Diseases, Neurology

## Abstract

Amyotrophic lateral sclerosis (ALS) is a lethal neurodegenerative disease that in most cases occurs sporadic (sALS). The disease is not curable, and its pathogenesis mechanisms are not well understood yet. Given the intricacy of underlying molecular interactions and heterogeneity of ALS, the discovery of molecules contributing to disease onset and progression will open a new avenue for advancement in early diagnosis and therapeutic intervention. Here we conducted a meta-analysis of 12 circulating miRNA profiling studies using the robust rank aggregation (RRA) method, followed by enrichment analysis and experimental verification. We identified miR-451a and let-7f-5p as meta-signature miRNAs whose targets are involved in critical pathogenic pathways underlying ALS, including ‘FoxO signaling pathway’, ‘MAPK signaling pathway’, and ‘apoptosis’. A systematic review of 7 circulating gene profiling studies elucidated that 241 genes up-regulated in sALS circulation with concomitant being targets of the meta-signature miRNAs. Protein–protein interaction (PPI) network analysis of the candidate targets using MCODE algorithm revealed the main subcluster is involved in multiple cascades eventually leads apoptosis, including ‘positive regulation of neuron apoptosis. Besides, we validated the meta-analysis results using RT-qPCR. Indeed, relative expression analysis verified let-7f-5p and miR-338-3p as significantly down-regulated and up-regulated biomarkers in the plasma of sALS patients, respectively. Receiver operating characteristic (ROC) analysis also highlighted the let-7f-5p and miR-338-3p potential as robustness plasma biomarkers for diagnosis and potential therapeutic targets of sALS disease.

## Introduction

Amyotrophic Lateral Sclerosis (ALS) is an adult-onset, fatal neurodegenerative disease characterized by selective and progressive loss of upper and lower motor neurons in the cerebral cortex, brainstem, and spinal cord^1^. Death ultimately occurs due to respiratory failure 2–5 years following the onset of symptoms, though a small portion of patients survives longer^2^. The heterogeneity in the phenotypes of ALS almost results in misdiagnosis, whilst early and accurate ALS diagnosis is crucial for ensuring quick access to available pharmacologic and therapeutic options and early care planning^3^. ALS may be inherited in 5–10% of the whole population of ALS patients (fALS), so more than 90% of ALS patients are sporadic (sALS), without any family history. The most frequent genes involved in the development of ALS are as follows: *C9orf72*, *SOD1*, *TARDBP*, and *FUS/TLS*^4^. Despite the identification of several causative genes, the underlying pathogenic mechanisms of ALS are not well understood up to now. Noteworthy, some ALS-associated genes, including *TARDBP* and *FUS*, encode RNA-binding proteins involved in miRNA processing^5^ and signify the role of miRNAs in the pathophysiology of ALS.

MiRNAs are small (18–25 nucleotides long), endogenous, non-coding RNAs that post-transcriptionally regulate protein-coding mRNAs^6,7^. The non-coding RNAs have been found to play a vital role in several diseases, including neurodegenerative diseases^8^. Recently, miRNAs are of great interest as a potential diagnostic and prognostic biomarker in several conditions. In terms of ALS, it is frequently reported that miRNAs participate in various molecular pathways associated with neurodegenerative development^2^.

Research barriers for identifying molecular pathways of ALS include small sample size, using monogenic animal models, or human tissue from autopsy at the end stage of neuron degeneration. Because access to nervous tissue in life is not ethically acceptable, a circulating molecular signature has been considered^9^. Pieces of evidence have shown circulating microRNAs expression can be used to monitor neurological diseases, including ALS^10^. Recently, some review studies analyzed researches that previously identified differentially expressed miRNAs in ALS patients^11–13^. Despite a large number of studies, systems level analysis has not been performed to ascertain ALS-related miRNAs. Indeed, there are multiple circulating miRNA profiling studies in ALS, but a poor overlap exists between miRNAs reported as deregulated in the high throughput screening studies^14^.

Here, to minimize the effect of heterogeneity among ALS patients and applied platforms, we performed a meta-analysis with systems biology approach via a robust method aggregating the results of studies comparing circulating miRNA expression profiles of ALS patients and controls over the 2012–2019 period. Also, we enriched the potential target genes to provide insights into pathological mechanisms of ALS. Furthermore, PPI networks were established to characterize essential genes that possibly correlate with ALS disease. Finally, we experimentally verified the results of the miRNA expression meta-analysis.

## Material and methods

**Figure 1:**
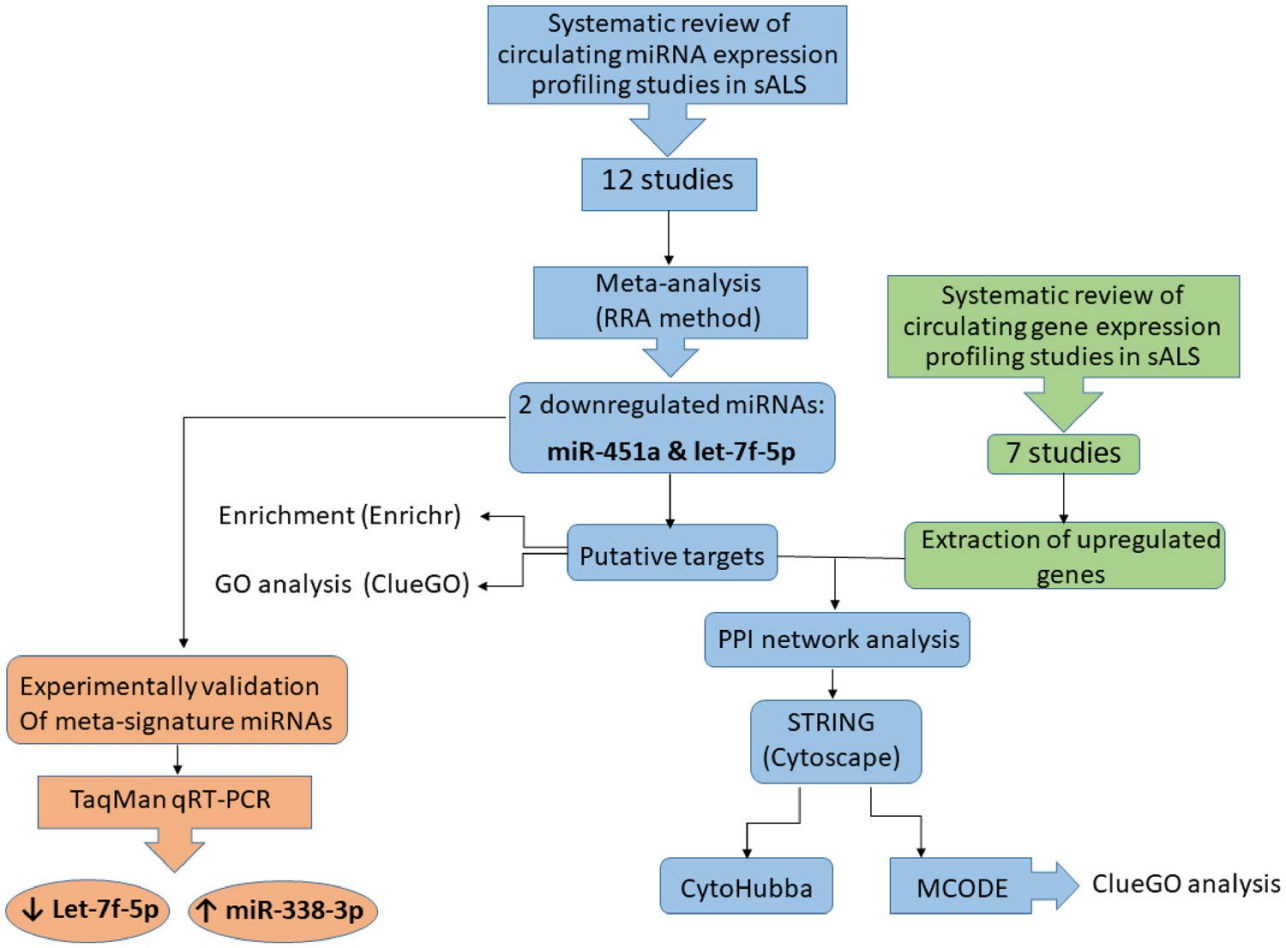
Overview of the study design.

The workflow for the overall procedure is shown in Fig. 1.

### Meta-analysis with systems biology approach

#### Literature search strategy

To identify miRNA expression profiling studies in ALS, we performed a search strategy as follows; we systematically searched the literature in PubMed (www.ncbi.nlm.nih.gov/pubmed/), Scopus (www.scopus.com), and EMBASE (www.embase.com) databases published up to 30 June 2020. We used search term TITLE-ABS-KEY ((miRNA* OR microRNA* OR mir-*) and (ALS OR Amyotrophic Lateral Sclerosis)) from article titles, abstracts, and keywords; by the way, we screened all references of relevant articles for additional studies. We also contacted the authors when the needed data were not available.

#### Eligibility criteria

We carefully assessed the titles and abstracts of each article and the full text of eligible studies. The inclusion criteria were: (1) original case–control studies in humans comparing the circulating miRNA expression between sALS patients and healthy control subjects; (2) employing miRNA expression profiling by using miRNA microarray, next-generation sequencing (NGS) or qRT-PCR; (3) reporting the separated lists of up- and/or down-regulated miRNAs according to respective cut-off criteria; (4) publishing in the English language. The exclusion criteria were as follow (1) studies that conducted on animal models or cell lines; (2) articles that evaluated only a few preselected miRNAs or a set of preselected miRNAs; (3) studies that did not focus on sporadic ALS, but just other types such as familial ones; (4) studies that evaluated miRNA expression in samples other than blood, such as muscles, brain and spinal cord; (5) studies that did not report complete lists of up- and/or down-regulated miRNA which were not available even after screening microarray datasets and contacting with authors; (6) review articles and case reports; (7) articles not reporting fold changes or *p*-value for differentially expressed miRNAs.

All full-text articles were carefully reviewed and the following items were collected from the eligible studies: first author, date of publication, the origin of study, number of samples, number of probes, profiling assay, and cut-off criteria. The lists of significant differentially expressed miRNAs were extracted from the studies. If the list (or full list) was not available in the article or Supplementary Appendices, we requested data. Where necessary, miRBase version 21 was used to obtain the standard name of miRNAs.

#### Robust rank aggregation analysis

We used “RobustRankAggreg” package in R software (available in the comprehensive R Archive Network website, http://cran.r-project.org/) to identify meta-signature miRNAs in sALS patients. The Robust Rank Aggregation (RRA) method normalizes the ranked miRNAs and assigns a significance score to each miRNA. The score is ranked according to ordering *p*-value so that the minimum *p*-value is assigned to the highest ranking. The probabilistic model makes the algorithm free and robust to outliers, noise, and errors^14^. Therefore, this method is a powerful and compelling approach for the identification of differentially expressed miRNA signature, and we utilized it for recording and ranking the lists of normalized up- and down-regulated miRNAs by using their fold change. Bonferroni correction was performed to reduce false-positive results.

#### Prediction of target genes of meta-signature miRNAs in sALS using online database

The R-based multiMiR package version 2.3.0 (http://multimir.ucdenver.edu) was retrieved to predict targets of meta-signature miRNAs. This package is a comprehensive collection of predicted and validated miRNA-target interactions and their associations with diseases and drugs. The multiMiR enables retrieval of miRNA-target interactions from 11 external databases to predict targets of miRNAs by TargetScan, DIANA-microT, ElMMo, miRDB, miRanda, MicroCosm, PicTar, and PITA databases. Furthermore, validated targets of miRNAs were retrieved from miRTarBase, TarBase, and miRecords databases. We searched the top 35% among all conserved and non-conserved target sites and selected only targets predicted by at least three algorithms or validated by one database for further analyses^15^.

#### Enrichment analysis of meta-signature microRNA target genes

In order to clarify the biological processes related to the identified targets, gene set enrichment was performed using Enrichr (http://amp.pharm.mssm.edu/Enrichr/) through its web interface. The Enrichr tool calculates *p*-values using a Fisher’s exact test and adjusts *p*-values using the Benjamini–Hochberg method to correct for multiple hypotheses testing^16^. In this study, gene set enrichment analyses were done against the following databases: Kyoto Encyclopedia of Genes and Genomes pathway (KEGG), Panther, and BioCarta enriched pathway. Further on, Gene Ontology (GO) analysis of meta-signature miRNA targets was conducted using the ClueGO version 2.0.7 app in Cytoscape^17^. ClueGO platform can analyze and visualize interrelations of terms and functional groups in biological networks based on the hyper-geometric distribution. In this study, *p*-value of < 0.05 and kappa coefficient of 0.4 was considered as threshold values. To correct the *p*-values for multiple testing, Bonferroni step-down was used.

#### Protein–protein interaction network analysis for microRNA target genes

PPI networks represent a model of interaction among gene pairs to explore disorder-related genes. In the present study, network analysis was performed using a more limited number of genes, whereby, we searched circulating gene expression profiling studies in sALS patients according to the criterion applied for miRNA expression profiling studies. Then, we extracted up-regulated genes and intersected the list with putative targets of meta-signature miRNAs.

PPI Network construction for selected genes was performed using the online search tool for the retrieval of interacting genes (STRING) database version 8^18^. Parameters were set to default. The network was subsequently imported into Cytoscape version 3.8 for further visualization and analysis. The Cytoscape plugin MCODE (molecular complex detection) version 1.6 was used to identify highly interconnected sub clusters of the PPI network using a degree cut-off of 2, a node score cut-off of 0.2, a K-core of 2, and a maximum depth of 100^19^. Then, topological analysis of constructed network was performed using CytoHubba plugin (version 0.1) of Cytoscape. to identify the essential/important nodes, so called hubs, by employing the 11 different algorithms as prescribed in^20^. Among all the algorithms, MCC (Maximal Clique Centrality) has a better performance in predicting essential nodes in the PPI network. The top 10 nodes ranked by MCC were employed to identify the sALS hub genes in this meta-analysis.

### Real-time quantitative reverse transcription (RT-qPCR)

Results obtained from the meta-analysis were validated by performing TaqMan Real-time qPCR in plasma specimens of sALS and control subjects. miRNAs are presented in the cell-free form of biofluids, including plasma. Plasma biomarkers are of interest because their detection is non-invasive and non-expensive. ALS patients without any family history of ALS and other neurodegenerative disorders were enrolled in this study. All patients and controls were evaluated by a neurologist to make sure that none of the controls had any neurological disease, and the patients had no neurological disorder other than ALS. All patients were neurologically evaluated using El-Escorial criteria and 30 patients with definite or probable ALS were selected. Also, 30 age- and sex- matched non-relevant healthy controls participated in this study. All affected and control individuals were informed of the nature of the research and consent form signed. This research was performed in accordance with the Declaration of Helsinki and with approval of the ethics board of the Iran University of Medical Science. Peripheral blood samples (3 mL) were collected from patients and controls under a fasting state and drawn into EDTA tubes. Within 3–4 h, the tubes were subjected to centrifugation at 200×*g* for 10 min at room temperature. Next, 1 mL aliquots of the plasma were transferred to 1.5 mL tubes and centrifuged at 10,000×*g* for 10 min at 4 °C to remove contamination or cellular debris. The supernatant was collected and stored at − 80 °C until use. The total RNA was extracted using Trizol reagent (Invitrogen). The corresponding cDNAs were synthesized using ExcelRT Reverse Transcriptase (SMOBIO) according to the manufacturer’s protocol. PCR amplification for TaqMan qPCR was performed using High-Rox 2 × Master Mix for Probe. RT-PCR reaction was conducted in a Corbett Rotor-Gene 6000 HRM Real-time PCR machine. The relative quantities of miRNAs expression in the cases and controls were calculated via the 2^−ΔΔCT^ method as “Fold change” values^21^. Each experiment was performed in duplicate. Since Fold change values had not normal distribution, the comparison of groups was performed using non-parametric Mann–Whitney U-test and *p*-value < 0.05 was considered as a significant cut off. Ultimately, receiver operating characteristic (ROC) analysis was performed to evaluate the diagnostic value of differentially expressed miRNAs. The area under ROC curve (AUC) implies the average value of sensitivity (true positive percentage) for all possible values of specificity (false positive percentage).

### Ethics approval

The study protocol conformed to the guidelines of the Ethics Committee of the Iran University of Medical Science (IR.IUMS.REC 1395.9221559202).

### Consent to participate

All patients and control subjects signed an informed consent form to participate.

### Consent for publication

All patients and control subjects signed an informed consent form to publish.

## Results

### Meta-analysis for finding meta-signature miRNAs in sALS

#### Literature search and included studies

The strategy used to include in this meta-analysis was demonstrated in the flow diagram (Supplementary Fig. S1). Briefly, 1446 possible eligible studies published between the years 2012 and 2019 were obtained in Pubmed, Scopus, and EMBASE databases considering our criteria. After eliminating duplicated publications, reviews, and unrelated studies such as studies considering ALS patients other than sALS and also the studies profiled non-circulating miRNAs, 99 articles met the eligibility. Finally, following full-text analysis, 12 articles used the whole miRNome profiling approach included in this meta-analysis^9,22–32^. Supplementary Table S1 provides the main information of each included study.

#### Meta-signature miRNAs in sALS patients

In our pooled datasets including 12 miRNA expression profiling studies, 219 miRNAs were reported to be differentially expressed in groups of 206 sALS patients and 181 healthy controls. Among 265 miRNAs, 167 were down-regulated and 98 were up-regulated. Furthermore, 29 miRNAs were reported in at least two studies. We applied the novel robust rank aggregation (RRA) method to identify consistently differentially expressed miRNAs across the profiles (Table 1, Supplementary Fig. S2).

**Table 1:** Meta-signature miRNAs in sALS patients.

miRNAs	p-value	Adjusted p-value	Count	References	Trend
**hsa-miR-451a**	2.15E−06	0.005549	4	^9,24,29,30^	Down
**hsa-let-7f-5p**	1.89E−05	0.048744	5	^21,22,26,28,30^	Down
hsa-miR-26b-5p	0.001058	1	3	^21,22,30^	Down
hsa-miR-16-5p	0.015687	1	2	^21,30^	Down
hsa-miR-183-5p	0.016547	1	2	^24,30^	Down
hsa-miR-26a-5p	0.022407	1	3	^21,28,30^	Down
hsa-let-7a-5p	0.026115	1	4	^21,25,28,30^	Down
hsa-miR-223-3p	0.02625	1	2	^29,30^	Down
hsa-miR-148a-3p	0.029116	1	2	^29,30^	Down
hsa-miR-338-3p	0.009681	1	2	^9,29^	Up
hsa-miR-34a-5p	0.011995	1	2	^24,28^	Up
hsa-miR-23b-3p	0.019227	1	2	^26,29^	Up
hsa-miR-4736	0.038918	1	1	^31^	Up
hsa-miR-224-3p	0.03907	1	1	^29^	Up

Significant values are in bold.

We ranked up- and down-regulated miRNAs by their fold changes. After using the RRA method, we identified 14 miRNAs (5 upregulated and 9 downregulated) with *p*-value < 0.05 among which, two downregulated miRNAs (hsa-miR-451a and hsa-let-7f-5p) were statistically significant meta-signature (Adj. *p*-value < 0.05).

#### Predicting the putative targets of meta-signature miRNAs

In silico analysis was performed to retrieve the putative target genes of hsa-miR-451a and hsa-let-7f-5p. The integration of eight predicted and three validated databases were systematically screened by using the multiMiR R package for miRNA–target interactions. 1,617 target genes were obtained according to our criterion. It’s noteworthy that all bioinformatic analyses were accomplished only for meta-signature miRNAs.

#### The enrichment analysis for putative target genes

**Figure 2:**
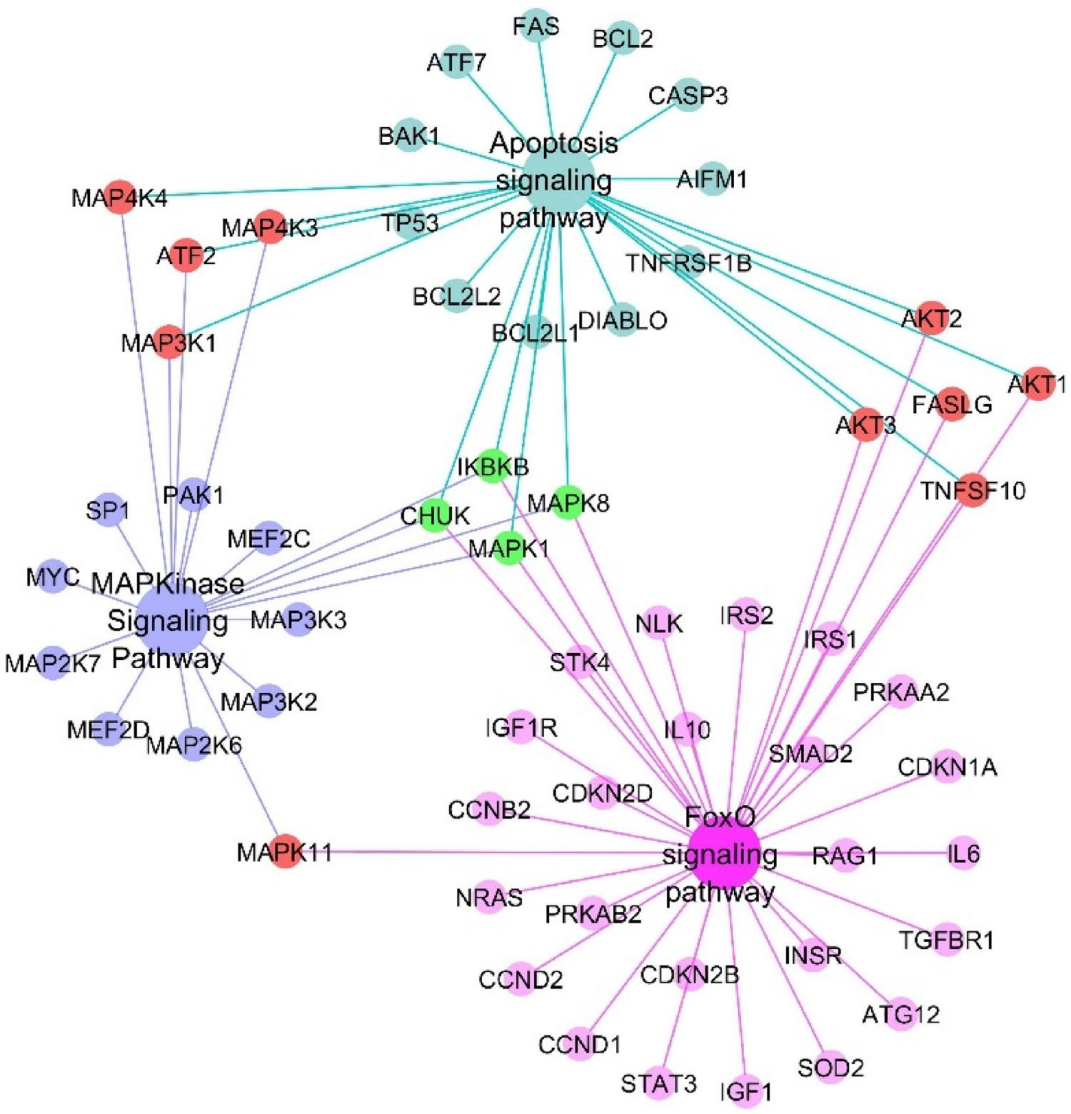
Multiple overlapping pathways identified using Enrichr analysis. Edges are colored based on their pathway of origin. Nodes colored in red and green are shared between 2 and 3 pathways, respectively.

Following the enrichment analysis by web-based Enrichr software, it was detected the most significantly enriched KEGG terms on sALS is “FoxO signaling pathway”. A KEGG pathway analysis also revealed 69 other pathways overrepresented in the putative targets of hsa-let-7f-5p and hsa-miR-451a, including “MAPK signaling pathway”, “PI3K-Akt signaling pathway”, “Amyotrophic lateral sclerosis” and “apoptosis”^33^. Panther 2016 displayed the meta-signature miRNAs target genes are mostly associated with “Apoptosis signaling pathway”. As well, BioCarta 2016 revealed a relationship between the “MAPKinase Signaling Pathway” and the predicted targets (Supplementary Table S3). Several databases analyzed by Enrichr (KEGG, Panther, BioCarta) revealed a relationship between the FoxO, MAPK, and apoptosis signaling pathways with the putative targets of hsa-let-7f-5p and hsa-miR-451a. Figure 2 demonstrates multiple overlapping pathways presented as the most significantly enriched terms in the three databases. *MAPK1/ERK2*, *MAPK8/JNK*, *CHUK/IKKa*, and *IKBKB/IKKb* are common genes in three pathways. The results of gene ontology enrichment through ClueGO revealed in terms of “biological process” and “molecular function”. Targets of let-7f-5p and miR-451a mainly involved in “protein serine-threonine kinase activity”, “anatomical structure morphogenesis”, “negative regulation of translation”, “cellular response to stress”, “metal ion binding” and “macromolecule metabolic process” (Supplementary Fig. S3).

#### PPI network construction and hub gene identification

**Figure 3:**
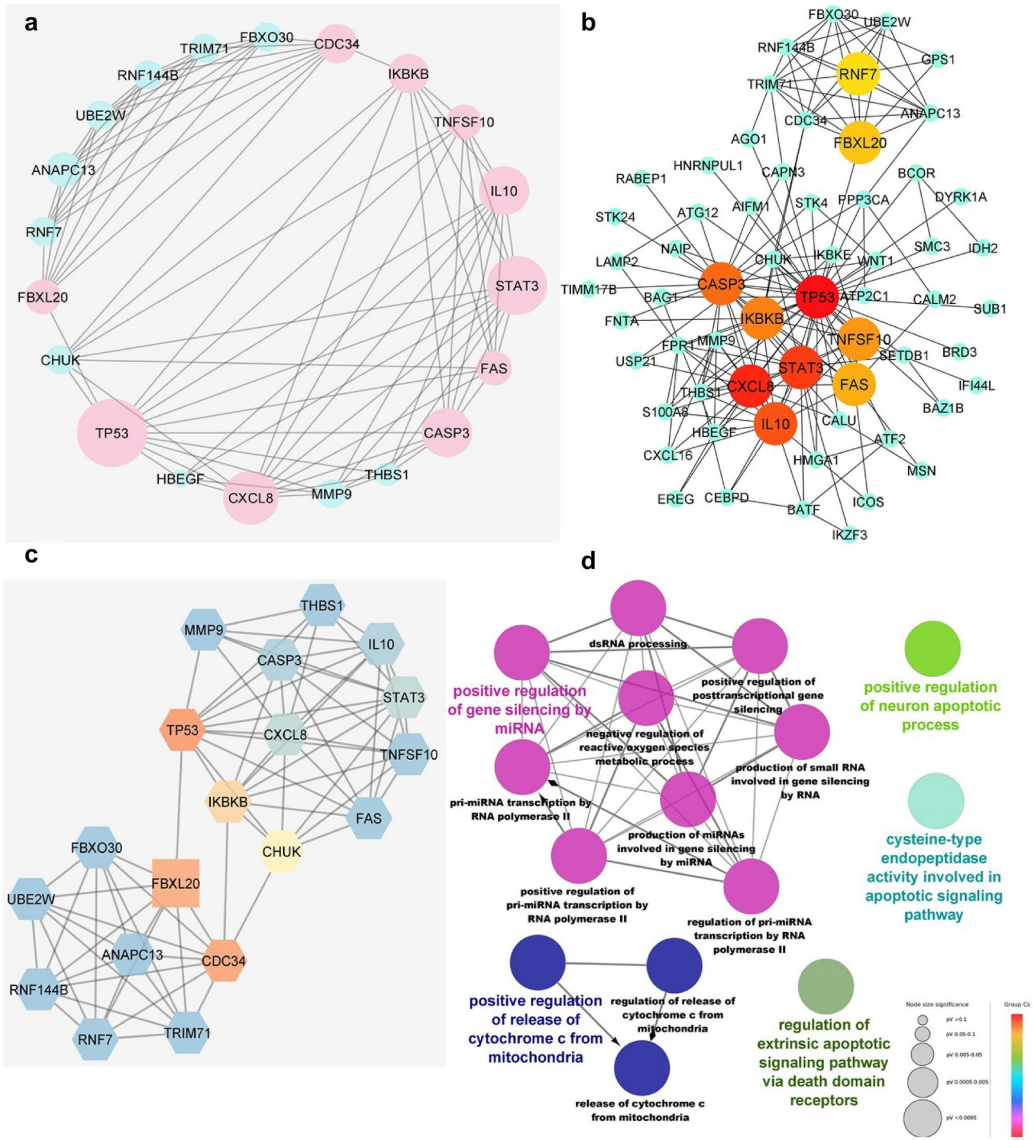
PPI network analysis of 241 candidate genes common with targets of meta-signature miRNAs. (**a**) network constructed using Cytoscape STRING database, degrees ≥ 10 (nodes colored in pink) were considered as hub genes. (**b**) The top 10 hub genes were selected based on the MCC algorithm in cytoHubba. The deeper the color of the node, the more significant the protein at the PPI network is. Nodes colored in green are the first stage nodes. (**c**) MCODE1 cluster is the most significant module picked up from the PPI network. Node shading is proportional to betweenness centrality; 0.25 by orange and 0 by blue. (**d**) ClueGO results of GO analysis of key proteins.

Since both differentially expressed meta-signature miRNAs are down-regulated, the targets are expected to be up-regulated. Therefore, we considered only up-regulated genes in the studies. By intersecting the putative targets of meta-signature miRNAs and the up-regulated genes in profiling studies, 241 candidate genes were selected to construct the PPI network using STRING as mentioned in the methods and material section. There were 338 edges and 238 nodes in the PPI network (PPI enrichment *p*-value: 0.000556, an average local clustering coefficient: 0.429, average node degree: 2.84). Cytoscape software was applied to visualize the interactive gene relationships. Degrees > 10 were considered the criterion of judgment. A total of 10 genes were identified as hub genes as follow: *TP53*, *STAT3*, *CXCL8*, *CASP3*, *IL10*, *CDC34*, *IKBKB*, *TNFSF10*, *FAS*, *FBXL20* (Fig. 3a). The top 10 hub genes were selected based on the MCC algorithm implemented in cytoHubba plugin (Fig. 3b). We further utilized the MCODE to detect clusters, which are highly interconnected regions in a network. By using MCODE, six network clusters (modules) were determined (Supplementary Table S5). The first cluster, MCODE1, with 19 nodes, 76 edges, and a cluster score of 8.444 was selected for GO enrichment and determining hub genes (Fig. 3c,d). The most significant GO terms over-represented by the hub genes associated with “positive regulation of gene silencing by miRNA”, “positive regulation of release of cytochrome C from mitochondria”, “cysteine-type endopeptidase activity involved in apoptotic signaling pathway”, “regulation of extrinsic apoptotic signaling pathway via DDRs (death domain receptors)” and “positive regulation of neuron apoptotic process”. The hub genes obtained from all three methods are largely common.

### Validation of the identified miRNAs

In order to verify the results of the meta-analysis, we evaluated the relative expression of miR-451a, let-7f-5p, and two top-ranked up-regulated miRNAs (miR-338-3p and miR-34a-5p) using TaqMan RT-qPCR in 30 sALS and 30 control subjects (16 men and 14 women in each group). The median age of patients and controls was 55.4 ± 12 and 54.4 ± 10 years, respectively. The detailed clinical features of participants are presented in Supplementary Table S6. According to the El-Escorial criteria (Supplementary Fig. S4), with the exception of four patients with clinically probable ALS, the rest were identified as clinically definite ALS patients.

**Figure 4:**
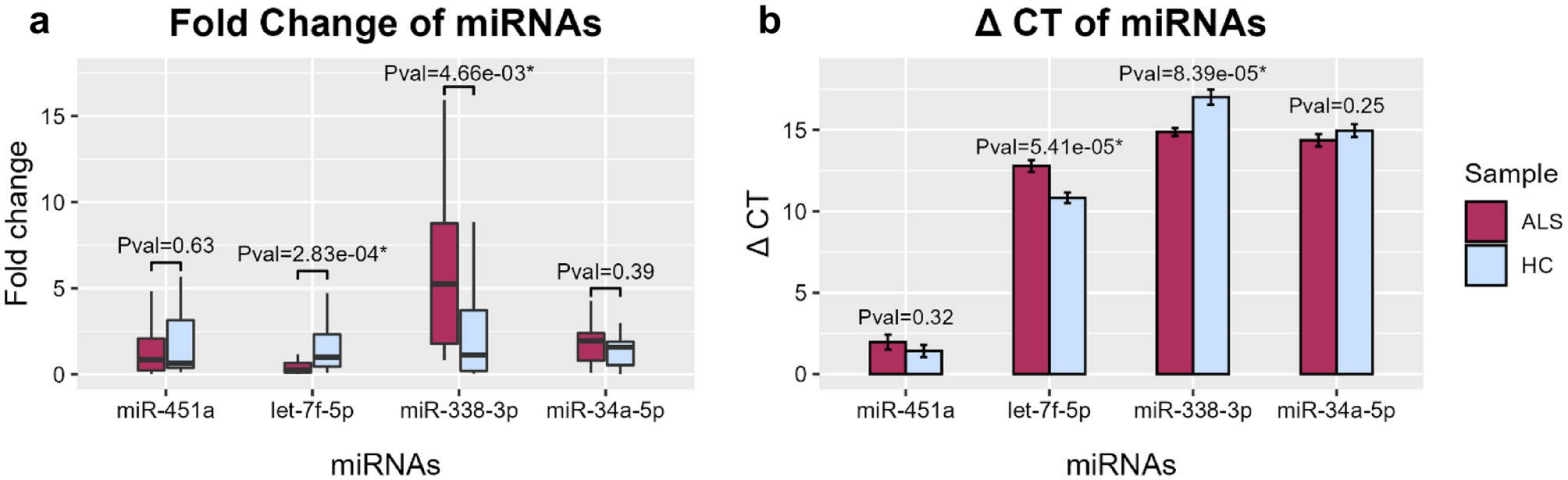
RT-qPCR analysis of miR-451a, let-7f, miR-338 and miR-34a levels in 30 ALS and 30 control. (**a**) The fold change of expression value (2^−∆∆Ct^) of miRNAs in ALS (gray) and control (white) normalized by the mean expression levels (∆Ct) of all controls. The boxplots are showing the median expression in ALS relative to control. (**b**) The CT of expression levels are presented as bar plots showing the error bars of miRNA expression in ALS and control.

Relative expression values were normalized to Cel-miR-39-3p as a housekeeping gene. The primers sequences were provided in Supplementary Table S7. RT-qPCR results demonstrated the expression of let-7f-5p was significantly downregulated (fold change = 0.25, *p*-value = 2.83e−4) in sALS patients comparing to healthy controls; whereas, miR-451a downregulated but not significantly (Fig. 4a,b). The miR-451a is erythrocyte specific, and its relative expression can be used as an indicator of hemolysis in serum and plasma samples^34^. Noteworthy, previous studies have revealed the fragility of red cells and a high degree of hemolysis in plasma samples from ALS patients^35^. Therefore, plasma and serum specimens are not appropriate for evaluation of erythrocyte-specific miRNAs such as miR-451, miR-16 and etc. in ALS disease. As presented in supplementary table s1, miR-451a was down-regulated in blood cells, therefore significantly differential expression in plasma was not observed. According to the results of experimental phase, miR-338-3p was significantly up-regulated (fold change = 4.38, *p*-value = 4.66e−3) in sALS patients relative to controls whereas, expression of miR-34a-5p was not significantly changed (Supplementary Table S8).

**Figure 5:**
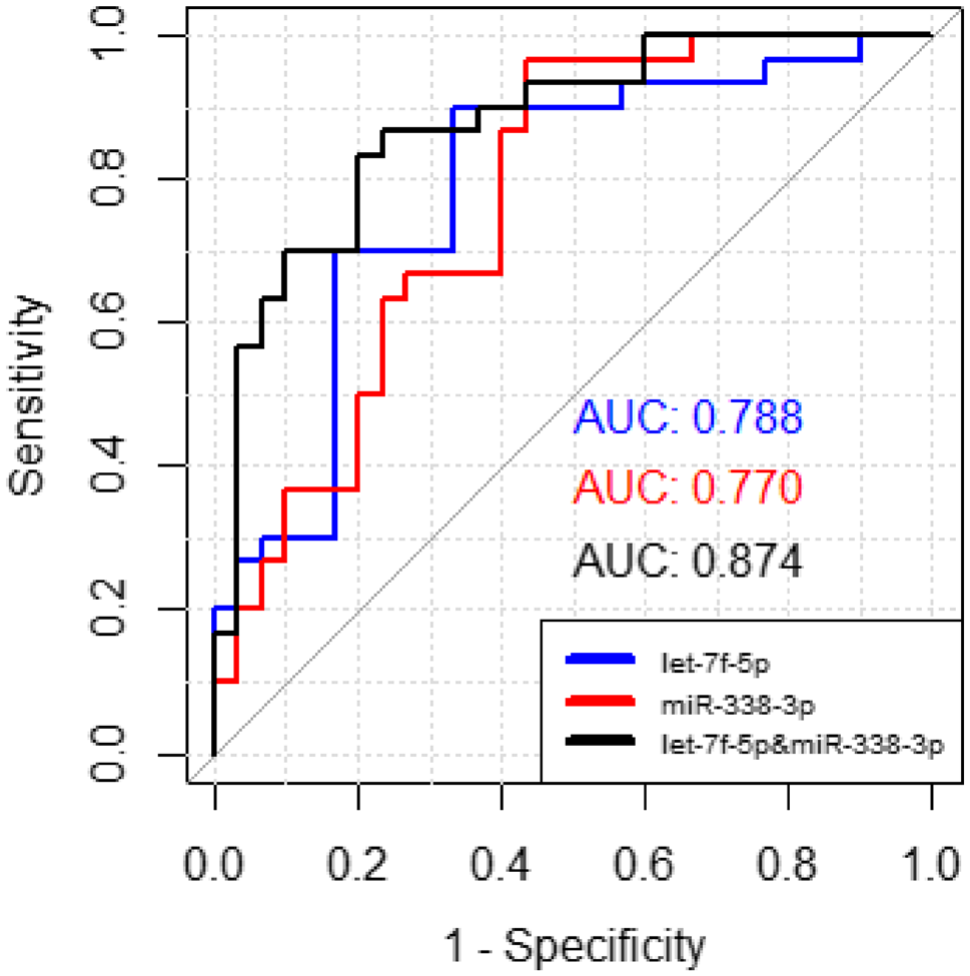
The ROC curve of let-7f-5p and miR-338-3p in distinguishing ALS patients and healthy controls.

ROC analysis was conducted to assess the sensitivity and specificity of the let-7f-5p and miR-338-3p as potential plasma biomarkers for ALS. The AUC values for let-7f-5p and miR-338-3p were calculated 0.78 and 0.77, respectively, highlighting the diagnostic efficacy of identified biomarkers. Further, ROC analysis with the combination of both miRNAs were performed and outcomes revealed the highest specificity with the AUC value of 0.87 (Fig. 5).

## Discussion

The identification of a molecular signature for amyotrophic lateral sclerosis is quite an intriguing yet challenging arena. Nevertheless, since obtaining tissue biopsies is the main limitation in studies of neurodegenerative diseases, tissue-based markers are not an applied option for early detection of ALS disease. Thus, this study aimed to explore circulating disease-specific markers that would be beneficial for diagnosis and treatment strategies in ALS. By targeting multiple transcripts and affecting the expression of numerous proteins, miRNAs play a crucial role in cellular functions^36^. Researches in miRNA expression patterns and high-throughput profiling have resulted in exploring novel biomarkers. However, limitations such as small sample size, using different platforms, various threshold criteria are some obstacles in getting a consensus to select miRNAs as biomarkers. To overcome the above-mentioned issues, a meta-analysis was performed by aggregating and comparing multiple ranked miRNA lists (RRA). In the present study, using the RRA method, two lists of up-regulated and down-regulated miRNAs have resulted according to the *p*-value. Among the miRNAs, two down-regulated miRNAs (hsa-miR-451a and hsa-let-7f-5p) were found to have significant diagnostic potential. To predict the functional impact of the identified miRNAs, their target genes were evaluated using systems biology analysis.

The gene encoding miR-451a, located in chromosomal region 17q11.2, plays a vital role in normal biological process and pathological condition. It acts as a tumor suppressor or oncomir in multiple cancer types^37^. It has been demonstrated that miR-451can protect cells against apoptosis induced via ischemia/reperfusion (I/R) injury and oxidative stress. MiR-451a overexpression causes to increase superoxide dismutase activity that leads to a decrease in neuron death^38^. Additionally, miR-451a protects erythrocytes from oxidative stress. Down-regulation of miR-451a causes accumulation of 14–3–3ζ that leads to inhibition of FoxO3 activity^39^. MiR-451a is also involved in the neuroinflammation process^10^. According to the present systematic review, miR-451a was down-regulated in leukocytes and peripheral blood of ALS patients^9,25,30,31^. As mentioned in the Results, miR-451a is degraded due to hemolysis and is not detectable in plasma of ALS patients, although the miRNA is altered in the circulation of these patients. Besides, downregulation of this miRNA is also reported as a blood biomarker in Parkinson’s disease (PD)^40^.

The let-7 family, as one of the first identified miRNAs, is highly conserved in sequence and function across species. They are known to be essential for development and viability^41^. In developing brain, let-7 promotes differentiation by suppressing genes of proliferation. The expression of let-7 decreases with advanced aging^42^. Deregulation of their expression causes several diseases including neurodegenerative disease, cancer, diabetes and etc.^43^. The human let-7 miRNA family comprises nine mature members with strong sequence similarity^41^. Let-7f-5p is located in the intergenic region at 9q22.3 and is reported to involve in a series of physiological and pathological processes^44^. Previous studies also have provided evidence of decreasing expression of let-7f in multiple sclerosis (MS) and Alzheimer’s diseases (AD)^44,45^. As presented in the systematic review, let-7f-5p expression was significantly downregulated in the peripheral blood, serum, plasma, and CSF of ALS patients^22,23,27,29,31^.

Functional enrichment analysis of miR-451a and let-7f targets revealed regulation via various pathways, including “FoxO signaling pathway”, “MAPK signaling pathway” and “apoptosis”. Apoptosis and MAPK signaling pathways are known in ALS^46^. Mitogen-activated protein kinases (MAPKs) are serine—threonine protein kinases that regulate various cellular activities including proliferation, differentiation, apoptosis, survival, and inflammation. Protein kinases have also been found to play key roles in several neurodegenerative diseases such as PD, AD, and ALS. MAPKs comprise c-Jun NH2- terminal kinase (JNK), p38 MAPK, and extracellular signal-regulated kinase (ERK), each of which exists in several isoforms^47^. In various signaling pathways, MAP kinases are involved as central incorporating. Proinflammatory cytokines and nitric oxide production in motor neurons and glial cells activate the p38 MAPK signaling pathway that plays a critical role in the propagation of environmental toxic among adjacent neuron cells, relatively rapid progression of ALS and eventually neuron death^48^.

Apoptosis mediated by intracellular processes, has been defined as programmed cell death. In the developing brain, apoptosis is of need for elimination of excess neurons, yet neuronal apoptosis is the main pathomechanism in various neurodegenerative processes^49^. Apoptosis may be initiated through one of intrinsic or extrinsic signaling cascades, albeit both dependent on caspases (cysteine proteases) function. Molecular pathways of apoptosis are activated in amyotrophic lateral sclerosis. According to research results, scavenging apoptosis delays neuronal death and prolongs survival in ALS models. Despite the various interfering, eventually, these models die, indicating that targeting the apoptosis cascade can slow the death trend but cannot abolish it^50^.

The FoxO family includes four transcription factors; FoxO1, FoxO3, FoxO4, FoxO6. Several upstream proteins regulate function and structure of FoxOs by post-translationally modification and affect subcellular localization and consequent transcription activity of them, i.e. they are downstream targets of the serine/threonine *PKB*/*AKT*. The *AKT* regulates processes related to cellular survival during neurodegenerative disorders. It is worth noting that *AKT* is a validated target of miR-451a. Downstream, FoxOs regulate the expression of various genes, afterward control several cascades, and eventually alter cellular function. In particular, FoxOs are involved in pathogenesis of neurodegenerative disease. Yet, FoxOs and their downstream targets have protective effects on neuronal dysfunction^51^. FoxOs, especially FoxO1 and FoxO3 are necessary for neuronal protection during aging. Strikingly, they are essential for neutralizing proteotoxic and oxidative stresses and preventing the accumulation of insoluble protein aggregates. In response to stress, FoxOs may either trigger neuronal death or survival cascades^52^.

Here, in circulating meta-analyzed studies, FoxOs were not part of differentially expressed genes, albeit their expression have altered in ALS spinal cords^53,54^. Additionally, recent studies have indicated potential roles for FoxOs in the pathogenesis of ALS. According to the results, the FoxO pathway is activated during ALS to protect against ALS related pathogenesis. Larger et al. found a significant up-regulation of FBXO32 in ALS skeletal muscles that correlate with a decreasing *AKT*. Although they saw no difference in FoxO1 and FoxO3 levels between patient and control groups, the results of the study implied that inhibition of *AKT* induces FoxO activity by decreasing cytosol sequestration^55^. Han et al. reported that a decrease in expression of neuronal vMSP, a cleavage product of *VAPB*, due to mutation of *VAPB/ALS8* in ALS, triggers FoxO activation, increases ATP levels, and prolongs survival in *VAPB* *C. elegans* mutants^56^. Zhang et al. have revealed in proteotoxic stress conditions; TDP-43 translocated to cytoplasm and relieved an inhibition on FoxO’s transcription activity in the nucleus. In cytoplasm, TDP-43 competes with FoxOs for binding to proteins 14–3–3 and consequently promotes the nuclear translocation and activation of FoxOs. The activation of FoxO transcription factors, including FoxO1 and FoxO3 induces autophagy, and in the case of FoxO3a, proteasome activity^57^. However, during chronic stress such as neurodegenerative trend, the potential of TDP-43 for defending the stress would be lost through lack of TDP-43 action resetting. Further, the over-activity of FoxOs led to cell death^51^. More recently, Watts et al. have reported suppression of MAP4K4 retains motor neuron viability by alleviating c-Jun induced apoptosis and FoxO mediated autophagy through decreasing protein aggregates in ALS^46^. On the other hand, activation of *MAP4K4*, a predicted target of let-7f, promotes degeneration of motor neurons. Phosphorylation of *MAP4K4* due to cellular stress leads to phosphorylation of JNK and then c-Jun, results in apoptosis^58^. Regarding motor neurons, it has shown FoxO3a play a role to determine stress response. In response to growth factor deprivation, *PI3K/AKT*, one of the central regulators of FoxO transcriptional activity, decreases. Then, FoxO3a translocate from cytoplasm to nucleus and induces apoptosis through activation of *Fas/FasL* and JNK pathways^59^. So though, the role of FoxO signaling pathway is not well specified in ALS disease, previous studies have revealed dysfunction of FoxOs is contributed to pathogenesis of ALS. Activated FoxO signaling may protect motor neurons against ALS associated degeneration or drive the neuron to death. So, it needs further investigations of FoxO interactions and identifying targets involved in protective effects. Many targets of the meta-signature miRNAs identified in present meta-analysis, have activities in the FoxO signaling pathway. As the importance of this molecular pathway in the ALS disease trend is becoming to be more explicit, altering in the expression of these miRNAs (miR-451a and let-7f-5p) can play a great role in controlling the ALS disease by affecting the FoxO signaling pathway.

PPI exhibits the interplay between the proteins in response to various disorders and has been shown that it is excellent helpful hints for identifying proteins that play an effective role in the disease^60^. In this study, a PPI network was constructed using STRING database. Applying the cytoHubba, the top ten hub genes contributing to ALS were predicted. Further, we used MCODE to discover the core module in the PPI network. The genes obtained from MCODE algorithm are mainly overlapped with cytoHubba network hub genes, including TP53, IKBKB, STAT3, CXCL8, FBXL20 that simultaneously have the strong connectivity in the STRING network. Ontology analysis of proteins involved in cluster1 indicates predicted target genes of let-7f and miR-451a, that meanwhile up-regulated in ALS patient circulation, are enriched in neuron apoptosis, gene silencing by miRNA, and multiple cascades of apoptosis. Our results provide independent support for findings obtained from meta-signature miRNA analysis and suggest that their targets involved in the PPI analysis are key factors in the pathogenesis of ALS. Although the results of RT q-PCR didn’t present significantly downregulation of miR-451a in the plasma of ALS patients. As described above, plasma is not an appropriate specimen for indicating miR-451a differential expression and the blood cells including leukocytes are better for detecting the difference. Besides these downregulated meta-signature miRNAs, two top-ranked miRNAs; miR-338-3p and miR-34a-5p, were experimentally verified in this study. The results demonstrated miR-338-3p is upregulated in plasma of sALS patients. The miR-338-3p, located at chromosome 17q25.3 was first reported in prion-induced neurodegeneration. Previous studies revealed the miRNA consistently upregulated in CSF, serum, leukocytes, and spinal cord of sALS patients^61^. Furthermore, it is reported that miR-338-3p was upregulated in neuromuscular junction of patients with sporadic ALS^30^. miR-338-3p is involved in several biological pathways that could contribute to ALS pathogenesis, including neurodegeneration and apoptosis. Several genes associated with ALS including *ARHGEF28* and *VAPB* are the targets of miR-338-3p. *ARHGEF28*, a novel ALS gene involved in the aggregation of light neurofilaments, is a validated target for miR-338-3p^62^. Also, a miR-338-3p predicted target, VAPB, is recently suggested as a diagnostic biomarker in ALS that complicated in ER-associated aggregates^63^. More recently, Song et al. reported FoxO3a induces miR-338-3p expression and may play a critical role in decreasing cell survival through directly suppressing the expression of NRP1^64^.

Finally, ROC analysis revealed that let-7f-5p and miR-338-3p show significant AUC value for sALS diagnosis. In addition, the combination of both miRNAs provided better performance in diagnosing ALS with higher AUC values.

## Conclusion

This study is the first meta-analysis with systems biology approach performed to find a consensus in identifying circulating miRNA biomarkers for sALS. We demonstrated that let-7f-5p downregulation and miR-338-3p upregulation are the potential circulating biomarkers for sALS. Certainly, they can be easily accessed from patient plasma. Further, bioinformatics analyses indicated that beyond the known pathways such as MAPK signaling and apoptosis, the FoxO signaling pathway is involved in ALS pathogenesis via multiple cascades.

## Supplementary Information

Supplementary Information.
