# Supplementary material for: Identification of let-7f and miR-338 as plasma-based biomarkers for sporadic amyotrophic lateral sclerosis using meta-analysis and empirical validation
Source: Sci Rep. 2022 Jan 26;12:1373. doi: 10.1038/s41598-022-05067-4 (PMC8791978; doi:10.1038/s41598-022-05067-4)
Supplement: Supplementary file 1 — Supplementary Information. [file 41598_2022_5067_MOESM1_ESM.pdf]

Supplementary File

Identification of let-7f and miR-338 as plasma-based biomarkers for sporadic Amyotrophic Lateral Sclerosis using meta-analysis and empirical validation

Narges Daneshafrooz, Mohammad Taghi Joghataei, Mehdi Mehdizadeh, Afagh Alavi, Mahmood Barati, Bahman Panahi Shahram Teimourian, Babak Zamani*

* Department of Neurology, Firoozgar Hospital, Iran University of Medical Science, Tehran, Iran

E-mail: zamba3@yahoo.com, zamaniba3@gmail.com

Supplementary Figure 1: Searching strategy

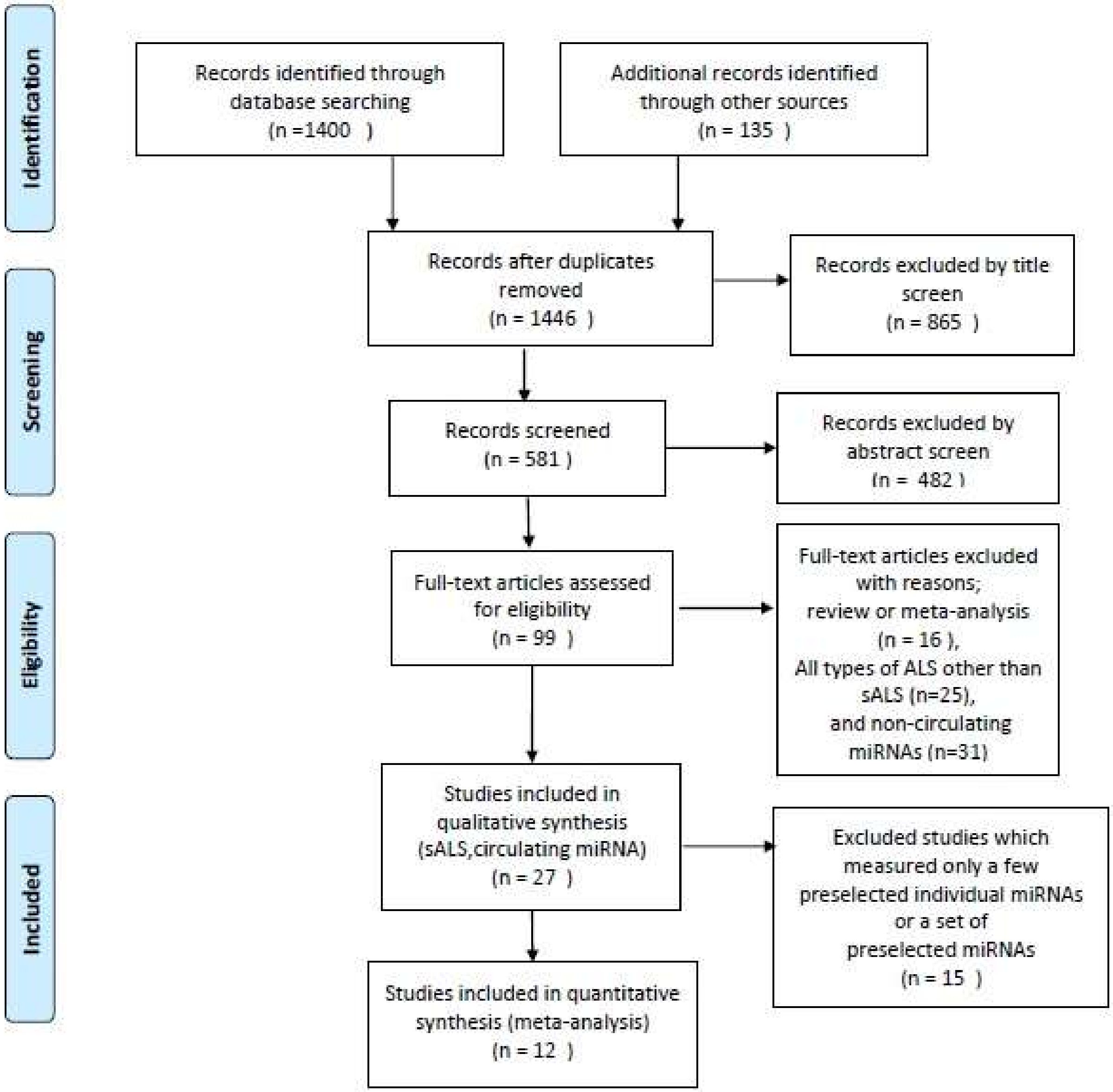

Supplementary Figure 2: Volcano map showing meta-signature miRNAs, resulted from meta-analysis. The miRNAs are plotted against their score ($\log_{10}$ p-value).

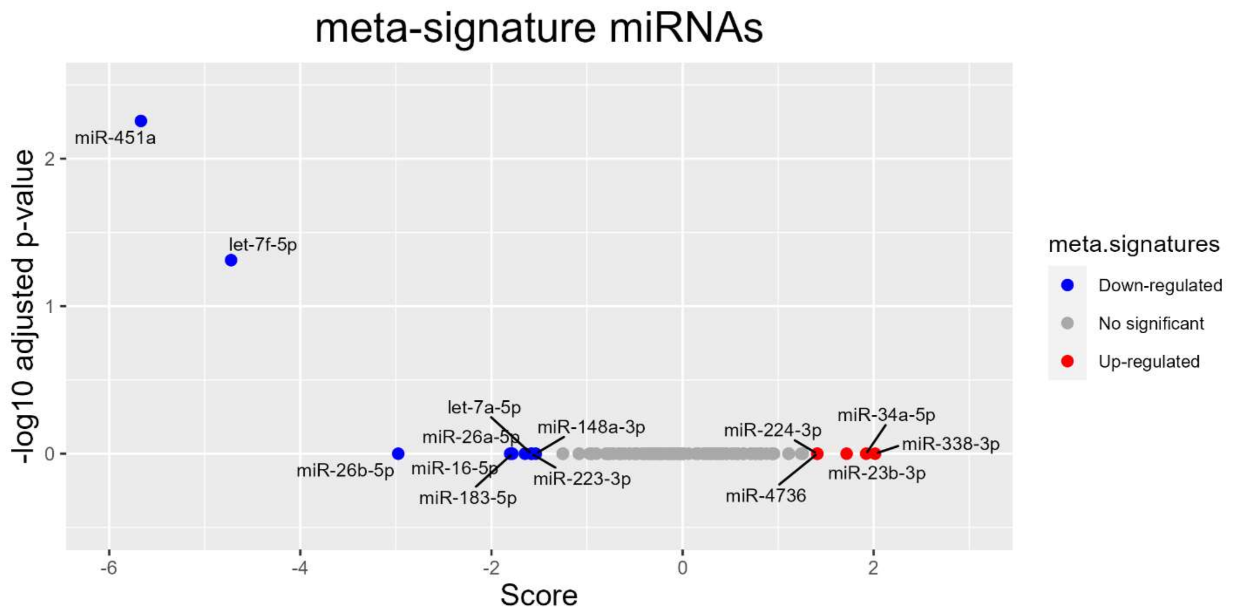

Supplementary Figure 3: Gene ontology analysis of biological process and molecular function for putative targets of hsa-miR-451a and hsa-let-7f-5p drawn using ClueGO.

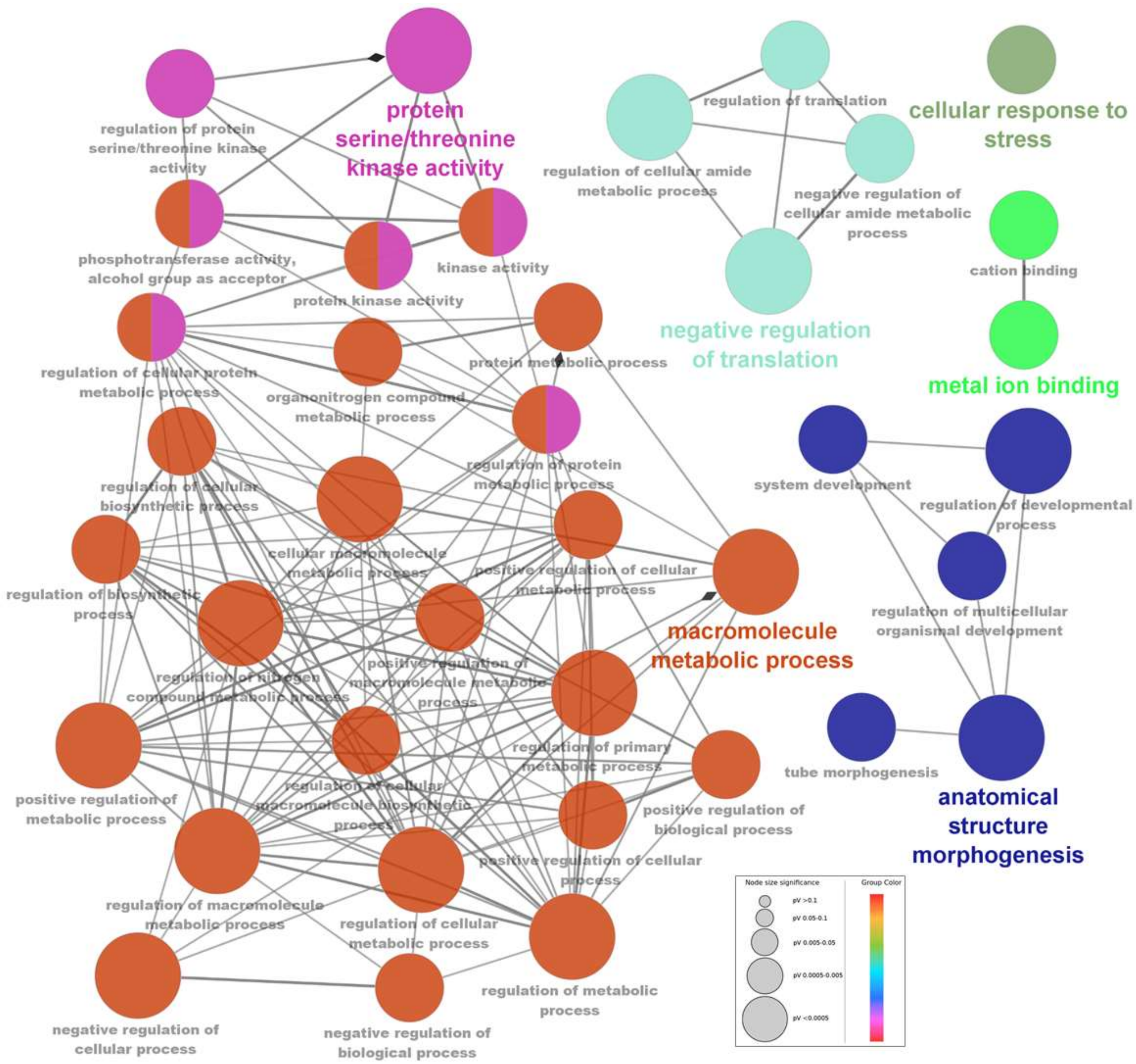

Supplementary Figure 4: The revised El-Escorial criteria for the clinical diagnosis of ALS (Gordon PH.2013).

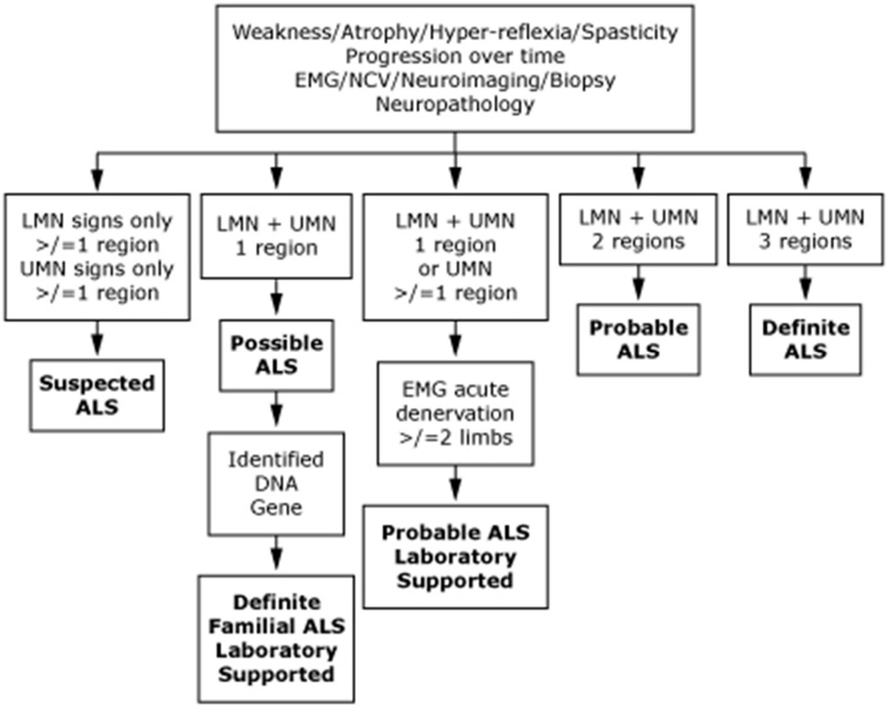

Supplementary Tables 1-8

Table S1. Characteristics of eligible miRNA profiling studies.

Table S2. The complete list of differentially expressed miRNAs extracted of 12 miRNA expression profiling studies. The miRNAs have been sorted by the number of repetitions in studies.

Table S3. Significantly enriched terms identified by Enrichr after querying of 1617 putative targets of hsa-miR-451a and hsa-let-7f-5p.

Table S4. Characteristics of eligible mRNA profiling studies.

Table S5. The network clusters detected by MCODE in the PPI network.

Table S6. Clinical features of sALS patients.

Table S7. The Primer sequences.

Table S8. RT-qPCR analysis for miRNA quantitation.

Supplementary Table S1

Author (year)	Reference number	country	No. of samples (sALS/HC)	Sample type	Assay type	No. of probes	Cut-off criteria
De Felice B (2012)	9	Italy	8/12	Blood leukocyte	miRXplore Microarray	911	>2 fold, Pval<0.0001
Butovsky O (2012)	21	USA	8/8	Blood monocytes	NanoString nCounter assay	664	>1.5 fold, Pval<0.05
Takahashi I (2015)	22	Japan	16/10	Plasma	3D-Gene® Human miRNA oligo chip	1800	>1 fold, Pval<0.05
Freischmidt A (2015)	23	Germany	18/16	Serum	Affymetrix miRNA 3.0 arrays	1733	>1.5 fold, Pval< 0.05
Chen Y (2016)	24	China	5/5	Blood leukocyte	GeneChip miRNA 3.0 Array	1733	>2 fold, Pval<0.05
Benigni M (2016)	25	Italy	16/24	CSF	human-miFinder 384HC miRNA PCR array	372	>1.5 fold, Pval<0.05
Waller R (2017)	26	UK	32/16	CSF	TruSeq small RNA sequencing	2585	Log2FC, Padj≤0.05
Waller R (2017)	27	UK	27/25	Serum	TaqMan Low Density Arrays	750	>1 fold, Pval<0.05
Raheja R (2018)	28	USA	20/30	Serum	LNA-SYBR-green-based RT-PCR	191	Pval<0.05
De Felice B (2018)	29	Italy	45/25	Periphral blood	Small RNA ION Proton Sequencer	2555	Lof2FC, Posterior probability≥ 0.8
Liguori M (2018)	30	Italy	6/5	Periphral blood	HiSeq2500 small RNA sequencing	2585	Log2FC, Padj≤0.05
Katsu M (2019)	31	Japan	5/5	Plasma external vesicles	3D-Gene Human miRNA Oligo Chip	2565	>1 fold, Pval<0.05

Supplementary Table S2

Down-regulated miRNAs	Count	Up-regulated miRNAs	Count
hsa-let-7f-5p	5	hsa-miR-1	2
hsa-let-7a-5p	4	hsa-miR-23b-3p	2
hsa-miR-451a	4	hsa-miR-338-3p	2
hsa-miR-26a-5p	3	hsa-miR-34a-5p	2
hsa-miR-26b-5p	3	hsa-miR-100-5p	1
hsa-miR-103a-3p	2	hsa-miR-101-3p	1
hsa-miR-106b-3p	2	hsa-miR-10b-5p	1
hsa-miR-134-5p	2	hsa-miR-1206	1
hsa-miR-142-5p	2	hsa-miR-1207-5p	1
hsa-miR-144-3p	2	hsa-miR-122-5p	1
hsa-miR-145-5p	2	hsa-miR-124-3p	1
hsa-miR-148a-3p	2	hsa-miR-126-5p	1
hsa-miR-151a-5p	2	hsa-miR-1260b	1
hsa-miR-15b-5p	2	hsa-miR-127-3p	1
hsa-miR-16-5p	2	hsa-miR-1296-5p	1
hsa-miR-183-5p	2	hsa-miR-1297	1
hsa-miR-21-5p	2	hsa-miR-133a-3p	1
hsa-miR-223-3p	2	hsa-miR-133b	1
hsa-miR-27b-3p	2	hsa-miR-137	1
hsa-miR-30b-5p	2	hsa-miR-144-3p	1
hsa-miR-320c	2	hsa-miR-144-3p	1
hsa-miR-340-5p	2	hsa-miR-144-5p	1
hsa-miR-423-3p	2	hsa-miR-149-3p	1
hsa-miR-425-5p	2	hsa-miR-153-3p	1
hsa-miR-93-5p	2	hsa-miR-15b-3p	1
hsa-let-7c-5p	1	hsa-miR-16-1-3p	1
hsa-let-7d-3p	1	hsa-miR-16-2-3p	1
hsa-let-7d-5p	1	hsa-miR-182-3p	1
hsa-let-7g-5p	1	hsa-miR-1825	1
hsa-let-7i-5p	1	hsa-miR-188-5p	1
hsa-miR-101-3p	1	hsa-miR-18a-5p	1
hsa-miR-106a-5p	1	hsa-miR-192-5p	1
hsa-miR-107	1	hsa-miR-193a-3p	1
hsa-miR-124-3p	1	hsa-miR-193b-3p	1
hsa-miR-1246	1	hsa-miR-194-5p	1
hsa-miR-1260	1	hsa-miR-19a-3p	1
hsa-miR-1268a	1	hsa-miR-19b-3p	1
hsa-miR-1274b	1	hsa-miR-20a-3p	1
hsa-miR-1275	1	hsa-miR-21-5p	1
hsa-miR-128-3p	1	hsa-miR-22-3p	1

hsa-miR-130a-3p	1	hsa-miR-224-3p	1
hsa-miR-130b-3p	1	hsa-miR-224-5p	1
hsa-miR-133a-3p	1	hsa-miR-24-3p	1
hsa-miR-135b-5p	1	hsa-miR-27b-3p	1
hsa-miR-139-5p	1	hsa-miR-29a-3p	1
hsa-miR-143-3p	1	hsa-miR-29b-3p	1
hsa-miR-143-3p	1	hsa-miR-29c-3p	1
hsa-miR-144-5p	1	hsa-miR-302c	1
hsa-miR-146a	1	hsa-miR-3194-3p	1
hsa-miR-146b-3p	1	hsa-miR-32-5p	1
hsa-miR-148b-3p	1	hsa-miR-326	1
hsa-miR-149-5p	1	hsa-miR-328	1
hsa-miR-150-3p	1	hsa-miR-331-3p	1
hsa-miR-150-5p	1	hsa-miR-339-5p	1
hsa-miR-151b	1	hsa-miR-33a-3p	1
hsa-miR-155-5p	1	hsa-miR-3607-5p	1
hsa-miR-15a-5p	1	hsa-miR-3619-3p	1
hsa-miR-182-5p	1	hsa-miR-371a-5p	1
hsa-miR-185-5p	1	hsa-miR-374b-5p	1
hsa-miR-186-5p	1	hsa-miR-379	1
hsa-miR-191-5p	1	hsa-miR-383	1
hsa-miR-1913	1	hsa-miR-421	1
hsa-miR-192-3p	1	hsa-miR-423-3p	1
hsa-miR-193a-5p	1	hsa-miR-4258	1
hsa-miR-195a-5p	1	hsa-miR-4298	1
hsa-miR-199b-5p	1	hsa-miR-4484	1
hsa-miR-19b	1	hsa-miR-4485-3p	1
hsa-miR-206	1	hsa-miR-4505	1
hsa-miR-20a-5p	1	hsa-miR-450b-5p	1
hsa-miR-214-3p	1	hsa-miR-455-3p	1
hsa-miR-22-3p	1	hsa-miR-4688	1
hsa-miR-221-3p	1	hsa-miR-4695-3p	1
hsa-miR-23a-3p	1	hsa-miR-4700-5p	1
hsa-miR-24-3p	1	hsa-miR-4736	1
hsa-miR-27a-3p	1	hsa-miR-4739	1
hsa-miR-28-3p	1	hsa-miR-513a-5p	1
hsa-miR-2861	1	hsa-miR-525-5p	1
hsa-miR-297	1	hsa-miR-548b-3p	1
hsa-miR-3064-5p	1	hsa-miR-548f	1
hsa-miR-30c-5p	1	hsa-miR-548g	1
hsa-miR-3176	1	hsa-miR-5684	1

hsa-miR-3177-3p	1	hsa-miR-580	1
hsa-miR-3187-5p	1	hsa-miR-592	1
hsa-miR-320a	1	hsa-miR-615-5p	1
hsa-miR-320b	1	hsa-miR-640	1
hsa-miR-328-5p	1	hsa-miR-649	1
hsa-miR-338-5p	1	hsa-miR-651	1
hsa-miR-342-3p	1	hsa-miR-660-5p	1
hsa-miR-34a-3p	1	hsa-miR-663b	1
hsa-miR-3605-5p	1	hsa-miR-877-3p	1
hsa-miR-361-5p	1	hsa-miR-9-5p	1
hsa-miR-3613-3p	1	hsa-miR-939-5p	1
hsa-miR-3690	1	hsa-miR-940	1
hsa-miR-374a	1	hsa-miR-99b-5p	1
hsa-miR-374b	1		
hsa-miR-378a-3p	1		
hsa-miR-3911	1		
hsa-miR-3935	1		
hsa-miR-3940-3p	1		
hsa-miR-424-5p	1		
hsa-miR-4299	1		
hsa-miR-4419a	1		
hsa-miR-4423-3p	1		
hsa-miR-4455	1		
hsa-miR-4507	1		
hsa-miR-4508	1		
hsa-miR-4538	1		
hsa-miR-454-3p	1		
hsa-miR-4646-5p	1		
hsa-miR-4674	1		
hsa-miR-4687-5p	1		
hsa-miR-4701-3p	1		
hsa-miR-4745-5p	1		
hsa-miR-4788	1		
hsa-miR-484	1		
hsa-miR-518d-3p	1		
hsa-miR-532-3p	1		
hsa-miR-532-5p	1		
hsa-miR-542-5p	1		
hsa-miR-550a-3p	1		
hsa-miR-551b-3p	1		
hsa-miR-574-3p	1		

hsa-miR-582-5p	1
hsa-miR-583	1
hsa-miR-584-5p	1
hsa-miR-595	1
hsa-miR-618	1
hsa-miR-634	1
hsa-miR-638	1
hsa-miR-652-3p	1
hsa-miR-664	1
hsa-miR-665	1
hsa-miR-7-1-3p	1

Supplementary Table S3

Term (KEGG)	P-value	Adjusted P-value
FoxO signaling pathway	1.29E-08	3.97E-06
AGE-RAGE signaling pathway in diabetic complications	2.60E-07	4.00E-05
MAPK signaling pathway	1.04E-06	1.07E-04
mTOR signaling pathway	3.96E-06	3.05E-04
Hepatitis B	6.15E-06	3.79E-04
p53 signaling pathway	1.20E-05	6.17E-04
Bladder cancer	1.23E-05	5.41E-04
Proteoglycans in cancer	1.23E-05	4.75E-04
Small cell lung cancer	1.29E-05	4.42E-04
Fluid shear stress and atherosclerosis	1.60E-05	4.94E-04
Adipocytokine signaling pathway	2.56E-05	7.17E-04
Relaxin signaling pathway	3.88E-05	9.96E-04
Signaling pathways regulating pluripotency of stem cells	4.50E-05	0.001067
PI3K-Akt signaling pathway	7.70E-05	0.001693
Pancreatic cancer	8.00E-05	0.001643
Autophagy	8.36E-05	0.001609
Kaposi sarcoma-associated herpesvirus infection	8.89E-05	0.00161
Chronic myeloid leukemia	9.54E-05	0.001633
Hepatitis C	1.16E-04	0.001878
Longevity regulating pathway	1.69E-04	0.002605
Neurotrophin signaling pathway	2.01E-04	0.002943
Glioma	2.72E-04	0.003801
Protein digestion and absorption	2.72E-04	0.003642

Cell cycle	3.67E-04	0.004713
Human papillomavirus infection	3.79E-04	0.004672
Colorectal cancer	4.59E-04	0.005441
Cellular senescence	4.75E-04	0.005418
Pathways in cancer	4.85E-04	0.00533
Adrenergic signaling in cardiomyocytes	5.87E-04	0.006233
Human cytomegalovirus infection	6.15E-04	0.006311
Focal adhesion	6.73E-04	0.00669
TGF-beta signaling pathway	7.93E-04	0.00763
Epstein-Barr virus infection	7.96E-04	0.007426
Type II diabetes mellitus	8.81E-04	0.007984
Prolactin signaling pathway	0.001257	0.011066
JAK-STAT signaling pathway	0.001297	0.011095
Chagas disease (American trypanosomiasis)	0.001446	0.012037
Measles	0.001609	0.013041
Melanoma	0.001671	0.013194
cGMP-PKG signaling pathway	0.001836	0.014134
Prostate cancer	0.001873	0.014073
Human immunodeficiency virus 1 infection	0.001878	0.013773
Non-small cell lung cancer	0.002138	0.015315
Progesterone-mediated oocyte maturation	0.002348	0.016435
Oocyte meiosis	0.002451	0.016777
TNF signaling pathway	0.003068	0.020545
Human T-cell leukemia virus 1 infection	0.003091	0.020256
Hypertrophic cardiomyopathy (HCM)	0.003153	0.02023
AMPK signaling pathway	0.003497	0.02198
C-type lectin receptor signaling pathway	0.003987	0.024562
Regulation of lipolysis in adipocytes	0.004042	0.024412
Amoebiasis	0.00422	0.024996
Gastric cancer	0.004257	0.024739
Transcriptional misregulation in cancer	0.004286	0.024446
Apoptosis	0.005482	0.030699
ECM-receptor interaction	0.005774	0.031755
Insulin resistance	0.005893	0.031845
Amyotrophic lateral sclerosis (ALS)	0.006934	0.036821
Insulin signaling pathway	0.007062	0.036863
GnRH signaling pathway	0.007473	0.038361
Breast cancer	0.007517	0.037954
Epithelial cell signaling in Helicobacter pylori infection	0.007826	0.038878
Fc epsilon RI signaling pathway	0.007826	0.03826
ErbB signaling pathway	0.007961	0.038314

Wnt signaling pathway	0.008517	0.040358
Hepatocellular carcinoma	0.008868	0.041386
Hippo signaling pathway	0.009825	0.045168
Lysosome	0.009994	0.045269
Influenza A	0.010884	0.048585

Term (BioCarta)	P-value	Adjusted P-value
MAPKinase Signaling Pathway	1.15E-06	2.74E-04
IL-2 Receptor Beta Chain in T cell Activation	2.18E-05	0.002587
Regulation of MAP Kinase Pathways Through Dual Specificity Phosphatases	3.28E-04	0.025911
FAS signaling pathway (CD95)	6.74E-04	0.026617
Overview of telomerase protein component gene hTert Transcriptional Regulation	6.12E-04	0.029021
NFAT and Hypertrophy of the heart	5.88E-04	0.034866

Term (Panther)	P-value	Adjusted P-value
Apoptosis signaling pathway	5.21E-06	5.84E-04
Interleukin signaling pathway	4.54E-05	0.002542
PDGF signaling pathway	7.95E-05	0.002968
p53 pathway feedback loops 2	1.74E-04	0.004863
Integrin signalling pathway	3.17E-04	0.007109
CCKR signaling map ST	7.66E-04	0.014289
p53 pathway by glucose deprivation	9.41E-04	0.015052
Insulin/IGF pathway-protein kinase B signaling cascade	0.001181	0.016539
p53 pathway	0.001452	0.018067
Oxidative stress response	0.002259	0.025298
EGF receptor signaling pathway	0.002771	0.028213
Ras Pathway	0.003224	0.030093
TGF-beta signaling pathway	0.004431	0.038174
T cell activation	0.005329	0.042635
B cell activation	0.005374	0.040126
PI3 kinase pathway	0.005593	0.03915
FGF signaling pathway	0.005735	0.037786

Supplementary Table S4

First author (year)	Sample number (ALS/HC)	Sample type	Platform	number of probes	cut off criteria
Saris CGJ (2009)	30/30	Perpheral blood	Illumina Sentrix HumanRef-8 Expression BeadChip	22185	FDR<0.05, FC> 1.01
Mougeot JLC (2011)	11/11	Lymphocytes	Agilent Human Whole Genome 4 × 44k Microarrays	45220	
Zhang R (2011)	20/20	PBMC	Affymetrix Human Genome U133 Plus 2.0 Array	45613	Pvalue<=0.0001, FC> 4
Zhao W (2017)	23/10	Monocytes	Illumina HiSeq1500	57736, Aligned with Hg19	Pvalue<0.05, FDR< 0.25, FC> 2
Linguori M (2018)	6/5	Peripheral blood	Illumina HiSeq2500	Alingned with Hg38	AdjPval<0.05, FC> 2, Number of reds> 25
Zucca S (2019)	4/4	PBMC	Illumina NextSeq 500		FDR<0.1, Log2FC> 1
Kentaro O (2019)	4/4	exosomes from cerebrospinal fluid (CSF)	Illumina NextSeq 500	Alingned with Hg38	

Supplementary Table S5

Cluster No.	Score	Node	Edge	Genes (Seed in red)
1	8.444	19	76	TP53, FAS, CHUK, IKBKB, UBE2W, THBS1, STAT3, CXCL8, IL10, TNFSF10, TRIM71, CASP3, ANAPC13, MMP9, FBXO30, FBXL20, RNF144B, CDC34, RNF7
2	4	5	8	DYNC1LI2, AHCTF1, SMC1A, BCOR, SMC3
3	4	4	6	CEP164, IQCB1, CEP57, CEP63
4	3	3	3	SNX17, MSN, RDX
5	3	3	3	LPGAT1, AGPAT6, AGPAT4
6	3	3	3	ANKRA2, ANKRD49, ANKRD46

Supplementary Table S6

	ALS patients	Controls
Number	30	30

Age[a] (years)	55.4 (34-65)	54.4 (31-62)
Sex (female:mail)	14:16	14:16
Initial symptom (spinal:bulbar)	20:10	NA
Disease duration (mounts)	34.48 (7-120)	NA
Diagnosis (definite:probable)	36:4	NA
ALSFRS-r score[ab]	21.5 (2-45)	NA

[a] Data are presented as median (range)
[b] Amyotrophic Lateral Sclerosis Functional Rate Scale revised score at the time of sample collection
NA, not applicable

Supplementary Table S7

Primer name	Primer sequence
451a RT	CGTCGTACTCAACGTGGTTAGGGTCCGAGGTATAGGTTCCCACGTGGAGGACGACGAACTCA
451a Forward	ACCGCGAAACCGTTACCATTA
451a Reverse	CGTGGTTAGGGTCCGAGGTA
451a Probe	FAM-CCCACGTGGAGGACGACGAACTCAG-BHQ1
Let7f RT	CGTCGTACTCAACGTGGTTAGGGTCCGAGGTATAGGTTCCCACGTGGAGGACGACGAACTAT
Let7f Forward	ACCGCGTGAGGTAGTAGATTG
Let7f Reverse	CGTGGTTAGGGTCCGAGGTA
Let7f Probe	FAM-CCCACGTGGAGGACGACGAACTATAC-BHQ1
miR-338-3p RT	CGTCGTACTCAACGTGGTTAGGGTCCGAGGTATAGGTTCCCACGTGGAGGACGACGCAACAA
miR-338-3p Forward	AAGCGTCCAGCATCAGTGAT
miR-338-3p Reverse	CGTGGTTAGGGTCCGAGGTA
miR-338-3p Probe	FAM-CCCACGTGGAGGACGACGCAACAAA-BHQI
miR-34a-5p RT	CGTCGTACTCAACGTGGTTAGGGTCCGAGGTATAGGTTCCCACGTGGAGGACGACGACAACC
miR-34a-5p Forward	AAGCGTGGCAGTGTCTTAGC
miR-34a-5p Reverse	CGTGGTTAGGGTCCGAGGTA
miR-34a-5p Probe	FAM- CCCACGTGGAGGACGACGACAACCA-HBQI
Cel-miR39 RT	CGTCGTACTCAACGTGGTTAGGGTCCGAGGTATAGGTTCCCACGTGGAGGACGACGCAAGCT
Cel-miR39 Forward	AAGCGTCACCGGGTGTAAAT
Cel-miR39 Reverse	CGTGGTTAGGGTCCGAGGTA
Cel-miR39 Probe	FAM-CACGTGGAGGACGACGCAAGCTG-BHQ1

Supplementary Table S8

miRNAs	$2^{-\Delta\Delta CT}$ value				ΔCT value			
	ALS	HC	U-test Pval	FC	ALS	HC	t-test Pval	ΔΔCT

**miR-451a**	1.79	2.33	0.63	0.77	1.97	1.42	0.32	0.68
**Let-7f-5p**	0.56	1.70	2.83e-04	0.33	12.79	10.83	5.41e-05	0.25
**miR-338-3p**	6.11	2.73	4.66e-03	2.34	14.88	17.01	8.387e-05	4.39
**miR-34a-5p**	3.17	2.08	0.39	1.52	14.36	14.95	0.25	1.51
